# Cancer and COVID‐19: Patients' and psychologists' reflections regarding psycho‐oncology service changes

**DOI:** 10.1002/pon.5461

**Published:** 2020-07-21

**Authors:** Chris Millar, Sophie Campbell, Peter Fisher, Jane Hutton, Andrew Morgan, Mary Gemma Cherry

**Affiliations:** ^1^ University of Liverpool Liverpool UK; ^2^ Liverpool University Hospitals NHS Foundation Trust Liverpool UK

**Keywords:** cancer, COVID‐19, psycho‐oncology, remote working

**Key Points**

1. The COVID‐19 pandemic has resulted in widespread disruption to oncology services
2. The Cancer Psychology Service at the Royal Liverpool University Hospital have changed their ways of working in light of these disruptions
3. In this letter, we reflect on psychologists' and patients' views of remote working
4. Patients and staff have generally adapted well to these changes, with high levels of patient engagement, despite some challenges
5. Remote therapy may continue to be useful for some patients even when face‐to‐face services resume

The Linda McCartney Centre is a patient‐centred cancer centre at the Royal Liverpool University Hospital, UK, offering a specialised psychology service for people experiencing significant distress related to cancer. We work as part of a multi‐disciplinary team within the cancer service and offer a range of empirically‐validated therapies. The team is comprised of six psychologists, several trainee psychologists and an assistant psychologist and received over 400 referrals last year from the North West of England.

Since the start of the COVID‐19 pandemic, we have changed our ways of working to continue to offer therapy, clinical supervision and staff training and support remotely, whilst complying with isolation directives. In this letter, we reflect on the themes that have emerged during our transition to remote working and our clinical practice. To inform this, we invited all psychologists within the services to complete a questionnaire asking about their experiences of remote working, and also asked them to nominate patient(s) from their caseload who may be willing to participate in an interview about their experiences. We also contacted patients who had declined therapy and opted instead to remain on the waiting list, to understand their experiences.

Watson et al^1^ note how patients decide if a mode of therapy delivery suits them by choosing whether to attend or not. Despite “teething difficulties” regarding home‐working dynamics, including lack of privacy, loss of non‐verbal communication, difficulties sharing formulation diagrams electronically, impracticalities of working with inpatients, and complications to behavioural work, high rates of engagement continued. To us, this is reassuring, and concurs with Cluver et al^2^ who observed that people with cancer reported strong positive perceptions and acceptance of therapy, regardless of whether service delivery was face‐to‐face or via videophone. Indeed, our patients have generally expressed surprise about the smooth transition to remote working. Several clinicians noted patients' relief at being able to access therapy without needing to return to somewhere associated with trauma—that is, the hospital where they underwent major surgery and intensive cancer treatment.

In terms of themes emerging during therapy, Cieślak^3, p.125^ notes how cancer can cause people to feel “internal chaos and uncertainty, to lose their sense of safety and experience a crisis.” In the current climate of COVID‐19, these may be familiar feelings within the public. Yet, when reflecting on our own clinical practice, we noted that patients have often already experienced significant life changes, meaning that the disruption from COVID‐19 does not always present entirely new experiences. Uncertainty, a lack of control, and concerns regarding risk of death are already present in the lives of many of our patients. Several patients have reflected to us that they feel better able to support friends and family to cope with the impact of COVID‐19 because of their experiences, and that their familiarity with managing uncertainty has meant that COVID‐19 represents another “what‐if?” to them.

Similarly, for some patients, infection control in the form of handwashing has always been of high importance, particularly for those patients who are immunocompromised due to their cancer or its treatment. Recently, the public message about hand‐washing and germ transmission has become common place. One psychologist reported that before the pandemic, their patients had experienced a lack of understanding from friends and family regarding their vigilance around good hygiene. They reflected on getting the message that they need to start living “normally” again, and were sometimes being called “OCD”. Now, as we are all being told about hand‐washing and germ transmission, these patients have felt a sense of validation that their focus was correct about the importance of the hygiene standards—even more so now.

Patients have also reflected how the pandemic has given them valuable perspective. One of our psychologists reflected that: “[patients] have reported being able to take a step back now from their cancer and to feel a sense of gratitude that they have received the treatment that they need and they are physically well. Some feel lucky that if they needed to go through cancer at all, that they were diagnosed and treated before all this happened and this is helping them to forge a sense of acceptance and gratitude around their cancer experience.”

It should be noted that some patients have decided that remote therapy may not or does not suit them, declining this and instead opting to remain on the waiting list until face‐to‐face appointments can recommence. To maintain engagement, psychologists arranged check‐ins with these patients on an individual basis. One patient, who declined continuing their course of therapy during COVID‐19, spoke of their concerns around being as honest in remote therapy as they would be in face‐to‐face therapy. They expressed concerns that this may give a false impression that they did not need psychological support. Another reported that, typically, they would usually be able to prepare for, and reflect on, therapy whilst driving to and from appointments, and mused the loss of this space. Yet others have raised concerns that their sessions are “being used up” managing the impact of COVID‐19 rather than addressing their initial cancer‐related goals. Their concerns may have implications for longer courses of therapy and create future challenges for service throughput more broadly.

COVID‐19 has shown we can continue to operate an effective service and provide support, albeit differently, by offering something rather than nothing to a very vulnerable population. Providing choice and catering for patients' preference of therapy delivery can ensure there are no disadvantages to their mental health benefits.^4^ As a team, we have been able to reflect that, when face‐to‐face working recommences, remote working may continue to be useful for some patients, such as those with limited mobility, or busy work or family commitments. Future comparisons with regards to pre and post COVID‐19 services may highlight interesting differences in routine outcome measures and rates of recovery—paving the way for novel considerations of resource allocation, given the as‐of‐yet unknown costs and benefits.

Chris Millar, Sophie Campbell, Peter Fisher, Jane Hutton, Andrew Morgan and Mary Gemma Cherry, on behalf of the Liverpool Cancer Psychology Service.

## DATA AVAILABILITY STATEMENT

Data sharing is not applicable to this article as no new data were created or analyzed in this study.
